# FunHoP: Enhanced Visualization and Analysis of Functionally Homologous Proteins in Complex Metabolic Networks

**DOI:** 10.1016/j.gpb.2021.03.003

**Published:** 2021-03-17

**Authors:** Kjersti Rise, May-Britt Tessem, Finn Drabløs, Morten B. Rye

**Affiliations:** 1 Department of Clinical and Molecular Medicine, NTNU – Norwegian University of Science and Technology, Trondheim NO-7491, Norway; 2 Department of Circulation and Medical Imaging, NTNU – Norwegian University of Science and Technology, Trondheim NO-7491, Norway; 3 Clinic of Surgery, St. Olavs Hospital, Trondheim University Hospital, Trondheim NO-7491, Norway

**Keywords:** Homologous proteins, Metabolic network, Pathway visualization and analysis, RNA-seq, KEGG, Cytoscape

## Abstract

**Cytoscape** is often used for visualization and analysis of metabolic pathways. For example, based on **KEGG** data, a reader for KEGG Markup Language (KGML) is used to load files into Cytoscape. However, although multiple genes can be responsible for the same reaction, the KGML-reader KEGGScape only presents the first listed gene in a network node for a given reaction. This can lead to incorrect interpretations of the pathways. Our new method, FunHoP, shows all possible genes in each node, making the pathways more complete. FunHoP collapses all genes in a node into one measurement using read counts from **RNA-seq**. Assuming that activity for an enzymatic reaction mainly depends upon the gene with the highest number of reads, and weighting the reads on gene length and ratio, a new expression value is calculated for the node as a whole. Differential expression at node level is then applied to the networks. Using prostate cancer as model, we integrate RNA-seq data from two patient cohorts with metabolism data from literature. Here we show that FunHoP gives more consistent pathways that are easier to interpret biologically. Code and documentation for running FunHoP can be found at https://github.com/kjerstirise/FunHoP.

## Introduction

Metabolic pathway analysis is a common framework for interpreting large-scale omics data and revealing functional trends and patterns in known biological multi-gene pathways. Important curated resources of metabolic pathways are the Kyoto Encyclopedia of Genes and Genomes (KEGG) [1], [2], Reactome [3], Panther [4], and similar knowledge bases [5]. Such resources are increasingly integrated with other knowledge bases, as can be seen for example for KEGG [6]. Several approaches can be used for analyzing metabolic pathways in the context of general network representations [7], and recent tools like eXamine [8] and Orthoscape [9] are relevant examples. For transcriptomics, an often-used approach is to map differentially expressed genes (DEGs) to known biological pathways, for example from KEGG. Such pathway representations can then be analyzed and visualized with commercial tools like Pathway Studio (www.pathwaystudio.com/) or iPathwayGuide (www.advaitabio.com/ipathwayguide.html), or free tools like CellDesigner [10] or Cytoscape [11].

**Figure 1:**
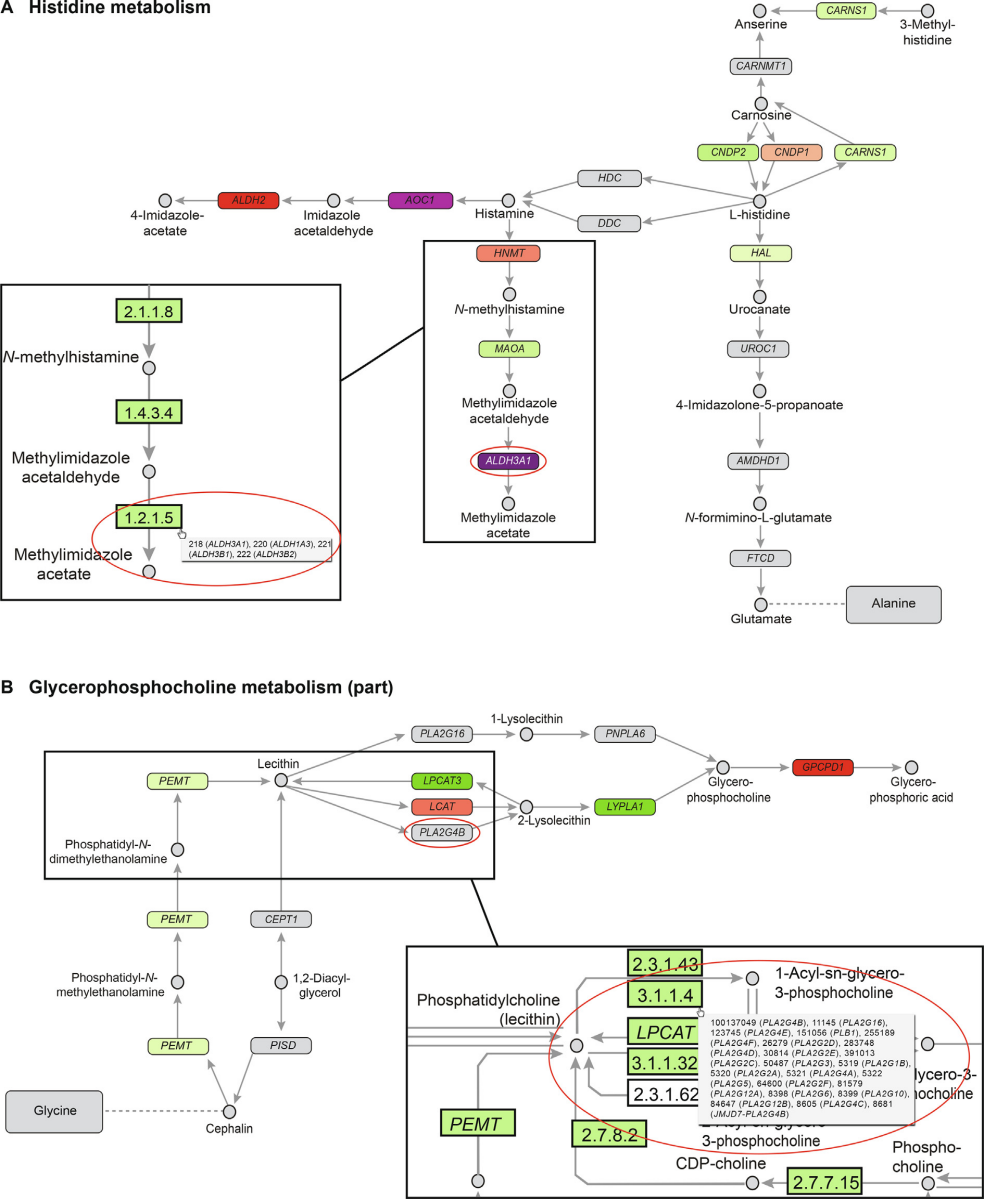
**Comparison of pathway XML files in Cytoscape to the same pathways in KEGG** **A.** A schematic of histidine metabolism pathway. All nodes in the original Cytoscape display show one single gene, including the *ALDH3A1* node. The *ALDH3A1* node from KEGG actually contains four genes: *ALDH3A1, ALDH1A3, ALDH3B1*, and *ALDH3B2*. **B.** A schematic of glycerophosphocholine metabolism pathway (part). The *PLA2G4B* node contains 21 genes, despite only showing one in KEGG.

In these tools, metabolic pathways are generally displayed as a network of metabolic transitions, where each transition is associated with a node representing the enzyme responsible for the transition. Each node typically represents a separate child from a structured pathway file, such as XML format. However, a challenge occurs when a transition from one metabolite to another can be catalyzed by more than one possible enzyme, *i.e*., by functionally homologous protein families, or functional homologs [12]. This is best illustrated by a typical example from KEGG. In the histidine metabolic pathway (KEGG: hsa00340), the four paralogs of NAD(P)^+^ dependent aldehyde dehydrogenase (*ALDH3A1*, *ALDH1A3*, *ALDH3B1*, and *ALDH3B2*, KEGG node index 1.2.1.5, Figure 1A) can all catalyze the transition from methylimidazole acetaldehyde to methylimidazole acetate. However, KEGG displays only the first gene, *ALDH3A1*, both in the website and in the XML file. In the website, the user can hover the mouse pointer over the gene in question to see any functional homologs, and the XML file does contain the KEGG IDs to all of them, although the corresponding gene names are not available in the file. In most conditions and cell types, one of these paralogs might be the preferred for the enzymatic transition, but in certain conditions one or several of the other three paralogs may become important, which should be taken into account. Though the selected example contains only four paralogs, the number of alternative enzymes can exceed 30 for some transitions, which complicates both visualization and interpretation of such nodes in the current framework. An example of a large node is the *PLA2G4B* node with 21 genes shown Figure 1B. In particular, the conclusion as to whether a node is overall up- or down-regulated will depend on the degree of differential expression of each gene (fold change and/or *P* value), the relative expression level of each gene in the node, and the enzymatic efficacy of the protein. The challenges regarding nodes with multiple genes are thus twofold. First, there is a need for data that can help us identify the most important enzyme(s) in conditions where multiple genes are able to perform the same reaction. Second, there is a need for improved visualization strategies to convey the relative importance of different enzymes with overlapping function when viewing biological networks from databases such as KEGG.

Cytoscape is a common tool for pathway visualization and analysis, often with data from KEGG. Pathways of choice can be downloaded from KEGG as KGML XML files (KEGG Markup Language, in XML format) and imported into Cytoscape using one of the many apps, such as KEGGscape [13]. In Cytoscape, the user can define styles, highlight nodes and/or edges, or change properties (*e.g.*, color, thickness, or shape of both nodes and edges based on uploaded data, such as gene expression or protein data). Layouts, statistical analyses, or specific apps with certain abilities can be applied to analyze the network in question. Importing the pathway is a crucial part of the analysis. The limitation in the KEGG XML files and/or KEGGScape of only showing the first of potentially multiple genes in each node has consequences for both analysis and interpretation (Figure 1), since the missing expression data of the remaining genes in the node makes it impossible to conclude on the overall gene expression associated with each node. It would be a huge advantage if one could expand the analysis to include differential expression of all genes in a node, and visualize the expression levels and associated differences for nodes consisting of multiple genes. This can be used to conclude on the overall up- or down-regulation at the node level, and suggest which gene(s) in the node that may have the largest influence on the overall activity.

Other options for importing KEGG XML files are CyKEGGParser [14] and CytoKEGG (http://apps.cytoscape.org/apps/cytokegg). CyKEGGParser discusses the topic of paralogs being grouped into single nodes, and their solution is to create new separate nodes for each of the genes within a multi-gene node. CytoKEGG is used to search and import KEGG pathways into Cytoscape. Dealing with multiple genes in the same node has also been discussed by others in a non-Cytoscape related context. The Bioconductor package Graphite [15] converts pathway topology to gene networks, and uses a combination of data from three curated databases (KEGG, Reactome, and BioCarta/NCI/NPID [16]) to create more complete networks. For the pathways from KEGG, Sales et al. [15] discuss how nodes with multiple genes may represent two different types of groups: protein complexes (“AND groups”, all genes should be considered together) or alternative proteins for the same function (functional homologs; “OR groups”, considering one gene at the time). This second group (OR) can be expanded into pathways without any connections between the alternative genes/proteins. In another publication, Wang et al. [17] acknowledge nodes with multiple genes by coloring the same node with multiple colors representing the different gene expression values. In addition, the number of genes in each node is displayed next to it. Although this approach can work for nodes with a limited number of genes, it will become harder to interpret when the number of genes increases. Additionally, neither of these approaches show the expression level for each gene, which can help to identify the genes that are most likely to be responsible for the reaction in a given node.

In nodes with multiple functional homologs, the relative expression levels of the genes in a node can be an accessible and useful measure to assess the relative importance of the individual enzymes for a given condition. For microarrays, the previous golden standard for gene expression analysis, differences in probe-affinities made it difficult to assess the relative expression levels between genes in an experiment [18]. However, the replacement of microarrays by RNA sequencing (RNA-seq) has now made comparison of expression levels feasible [18], [19], [20], [21]. Data from RNA-seq could therefore be utilized to improve the analysis of the overall node activity, as well as the individual contribution of each gene in the node for a given metabolic pathway.

Here we present Functionally Homologous Proteins (FunHoP): a method to improve gene expression pathway analysis and visualization. FunHoP improves the network visualization and analysis with respect to differential expression of nodes with multiple genes, and the relative contribution of each gene in a node. In particular, FunHoP aggregates gene information for each KEGG node consisting of multiple genes by using RNA-seq gene expression data for each gene, assuming that genes in the same node represent overlapping enzymatic potential (*i.e*., functional homologs). We show that prioritizing genes based on read counts from RNA-seq will improve the interpretation of differential expression results when analyzed with KEGG metabolic pathways. By gaining information from multiple genes for each node as input for differential expression analysis, we receive more biologically relevant and reliable pathways. Using prostate cancer (PCa) as a model system, we present two case studies showing how gene expression data are able to explain previously observed metabolic changes when FunHoP is applied.

## Method

RNA-seq data for PCa (read counts and gene identifiers) were downloaded from The Cancer Genome Atlas (TCGA) [22] at https://portal.gdc.cancer.gov/repository. For the Prensner cohort [23], RNA-seq raw reads in *fastq*-format were downloaded with approval from The database of Genotypes and Phenotypes (dbGap: phs000443.v1.p1, project No. 5870) at https://www.ncbi.nlm.nih.gov/projects/gap/cgi-bin/study.cgi?study_id=phs000443.v1.p1.

Raw RNA-seq reads were mapped to the hg19 transcriptome using TopHat2 [24], and featureCounts [25] was used to assign the reads to each gene. Voom [26] was further used for differential expression analysis. DEGs with a *P* value below 0.05 were extracted, and *P* values were log_2_ transformed by:

(1) $$\text{Value}={\text{log}}_{2}\;\;P\;\;\text{value}\times \left(-10\right)\times \left(\text{regulation}\right)$$

where regulation was defined as 1 for up-regulated genes (positive fold-change) and −1 for down-regulated genes (negative fold-change). Average RNA-seq read count for each gene was calculated using the mean of the two average values calculated over cancer and normal samples, respectively. All read counts were adjusted for gene lengths by a factor estimated by taking the gene length of the respective gene (sum of exons) divided by the average gene length over all genes.

In this study, 85 pathways of relevance to human metabolism, from subcategories 1.1 up to and including 1.11, were downloaded with human genes from the KEGG pathway database [27]. 71 of these did not contain any “line” nodes, and were used further (see Tables S1 and S2). The initial pathway analysis was performed by loading original KEGG XML files into Cytoscape (v. 3.4.) via the KEGGScape app and using a color gradient based on differential expression. All displays of differential expression used the same gradient: values were found on a scale from −1200 (black) to 600 (dark green), via −600 (purple), −300 (bright red), 0 (light yellow), and 300 (bright green). All values below zero showed down-regulated gene expression, and all values above zero showed up-regulated gene expression.

To expand the XML files to show all the genes in all the nodes, the list of human IDs and corresponding gene names was downloaded [28]. Using the ElementTree XML API, name strings in nodes with more than one gene were extended to include only the human names for all these genes (File S1). KEGG's solution to protein complexes was used as a base, and nodes with more than one gene were expanded. The expanded nodes were made by creating a new child for each of the genes that were not included in the initial child, and combining the new children along with the old child in a common node. The gene nodes use the same coordinates as the original gene, making it appear in the same place. To distinguish gene nodes from protein complexes, the gene nodes were made bigger than the default size, giving them a white field on each side. Differential gene expression was first used in combination with the expanded networks, showing how all the genes in the pathway were expressed.

To make more interpretable networks yet containing all the information, all genes within a node were aggregated into one. Name strings were extracted from all the network files, and the lists of unique names were defined as unique nodes. These included both single-gene and multiple-gene nodes. All gene nodes were named on the form “gene1-Bx”, where “gene1” is the name of the first gene in the gene-name string for a given node, and “x” is the number of genes within the node. For single-gene nodes x is 1. The total read count for a node was found by adding the read counts for all genes in the node, and this value was used for differential expression analysis at the node level. The contribution of each gene to expression level within a node was calculated as the fraction of the read count for that gene to the total read count of the node. The read count for each gene was used to style for expanded networks according to the relative expression levels of the genes. Nodes were colored on a scale from 0 to ≥ 50,000 read counts, changing from white to dark blue via shades of pink and blue.

The aggregated network files were adapted to work with the aggregated network gene node names. Changing the name strings to reflect the first gene name and the number of genes made the string similar to the gene node name format, and the network files could again be used together with the output from differential expression. The previously used style for differential expression was again used for the aggregated networks.

To show that the method works, two case studies were performed: the histidine metabolism pathway and a minor part of the metabolic pathway for glycerophosphocholine (GPC). The original files from KEGG were run through FunHoP’s steps of creating expanded and aggregated networks, as explained above, analyzed with differential expression, and visualized as expanded networks at the gene level and aggregated networks at the node level.

## Results

### Expanding nodes and using RNA-seq counts to improve pathway analysis

**Figure 2:**
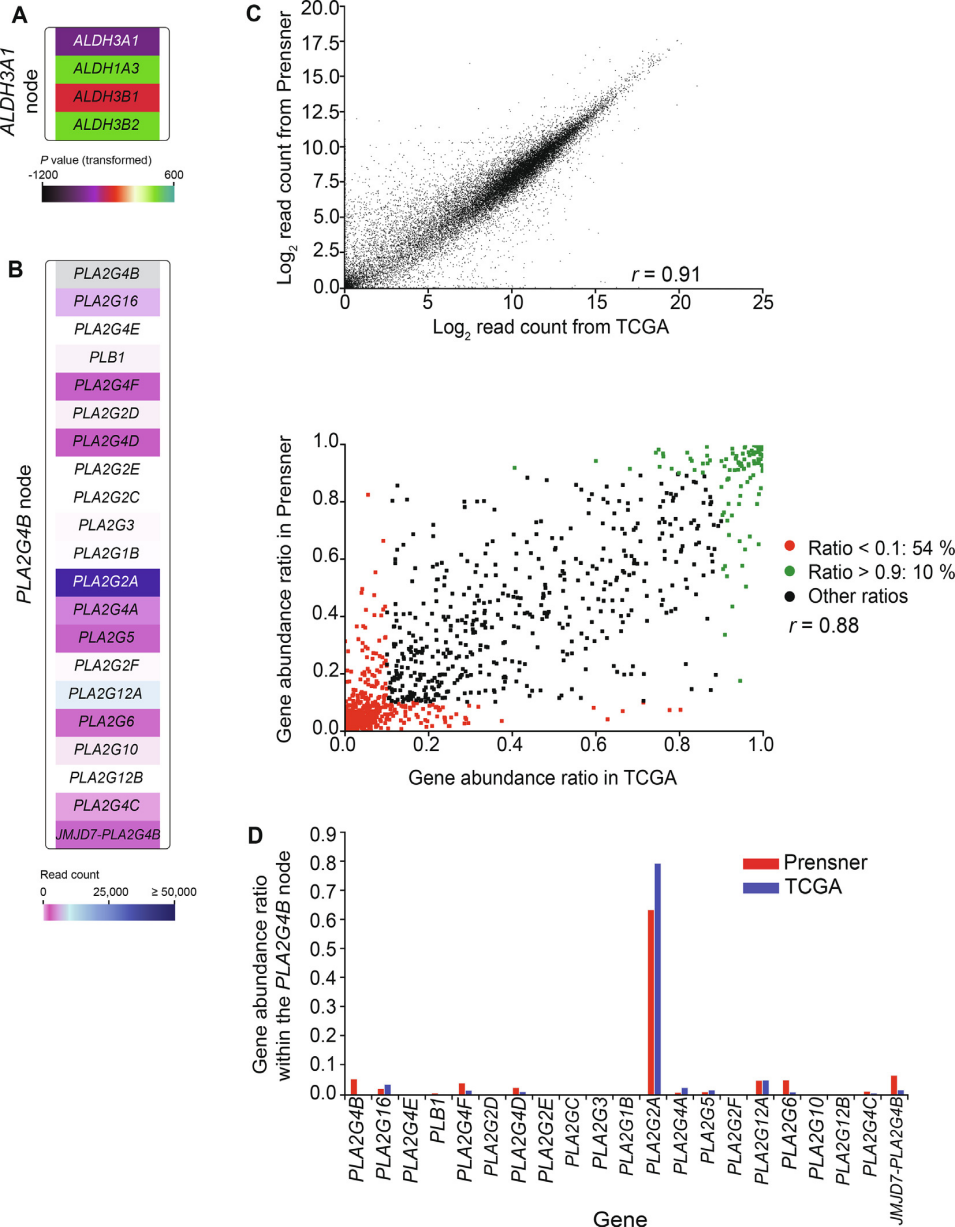
**Validation of the FunHoP approach** **A.** The *ALDH3A1* node expanded to show individual genes styled by differential gene expression. **B.** The expanded *PLA2G4B* node with its 21 genes. The *PLA2G4B* gene itself is not found in the dataset, leading to the whole node to be seen as not significant when only *PLA2G4B* is shown, although the other hidden genes are significantly differentially expressed. **C.** Plots of log_2_ read count from TCGA and Prensner (top) and gene abundance ratio in TCGA *vs*. in Prensner (bottom). **D.** Gene abundance ratios within the *PLA2G4B* node from (B) are comparable between the TCGA and Prensner cohorts.

To visualize KEGG pathways using information from all the individual genes involved, each node containing multiple functional homologs was expanded to show all genes in the node. Nodes were expanded by adding a new child for each gene belonging to the node, in addition to the existing child representing the default gene displayed in the pathway from KEGG in Cytoscape by KEGGScape. Old and new children of a node were then connected in a type “group” child, using the same strategy as for protein complexes (AND groups). To visually distinguish nodes with functional homologs (OR) from protein complexes (AND), the nodes were made bigger than the default size, giving them a white border on each side (Figure 2A).

We then used the average RNA-seq read count for each gene (normalized against gene length), generated from patient samples in two available PCa cohorts [22], [23]. Aggregated average read counts for all genes in each node were used to define the total expression level of each node in the network. Moreover, the relative read count for each gene in a node divided by the total read count for the node was used to define the relative expression contribution from each gene in a node.

To show the effect of FunHoP, original pathways were color-coded according to log-transformed *P* values from differential gene expression analysis, here comparing PCa tissue with normal prostate tissue. For showing individual genes within an expanded node, each gene was color-coded by both *P* values and the average read count for the gene to indicate expression level, giving two expanded networks that were comparable. The final representation shows the network with aggregated nodes, color-coded by differential expression based on overall read counts within a node.

### Assumptions regarding gene families and expression levels

We introduce two important assumptions for the biological interpretations in FunHoP. First, we observe that genes assigned to the same node usually belong to the same functional gene family or are closely related, as in the case of the nodes for the aldehyde dehydrogenases (Figure 2A) and phospholipases (Figure 2B). Thus, we make the assumption that the gene products also have similar function, in particular that they are able to catalyze the same main reaction, and describe them as enzymes with homologous function, or functional homologs. Therefore, we assume that each homolog can catalyze the reaction at a comparable rate. This is obviously an oversimplification, but also a necessary simplification given the general lack of rate data for most cellular processes.

Second, we assume that read counts from RNA-seq are indicative of the relative expression level of genes within a sample cohort. To check this assumption, we used RNA-seq read counts from two independent datasets. We see that the gene expression levels based on RNA-seq read counts are highly correlated (Figure 2C). We also find that expression ratios for individual genes in a node are correlated (Figure 2D). In particular, there is a very good correspondence for genes having particularly high (> 0.9) or low (< 0.1) ratios, which shows that RNA-seq data can robustly identify genes with a very high or very low relative abundance. This pattern is also evident when looking at individual genes within a node with high complexity, as the ratio for each gene within the node follows the same trend independent of which dataset we used (Figure 2D). The highly expressed *PLA2G2A* is clearly dominant in both datasets, the genes with very low number of read counts are the same, and the genes identified with few and intermediate number of read counts are also the same, though the relative ratios vary somewhat among the intermediate genes in the two datasets.

Under these assumptions, a gene’s contribution to the overall node activity is proportional to its expression level. This information becomes particularly useful in situations where one specific gene is dominating within a node. An example of this is the *PLA2G4B* node in the glycerophospholipid metabolism pathway (KEGG: hsa00564). The current Cytoscape/KEGGScape/KEGG framework only shows *PLA2G4B*, which is not found in the TCGA dataset, and hence the node seems to be not significant in the pathway. When the node is expanded, we see all 21 genes or functional homologs. By comparing the read counts for each gene, we see how *PLA2G2A* is expressed at a level that is ten times higher than the second one on the list (Figure 2B). Here, the darkest blue corresponds to ≥ 50,000 read counts, whereas the white/pink/light blue corresponds to < 5000 read counts. The genes indicated in light pink have < 10 read counts, and the ones in white are not expressed. These genes will most likely not contribute significantly to the pathway in this case. The KEGG default gene *PLA2G4B* is not found in the TCGA dataset, and has a low expression in the Prensner dataset. In this case, it is reasonable to assume that *PLA2G2A* is the main driving force for the transition represented by the node.

### Case studies

To investigate the impact of FunHoP on real biological interpretation of networks, we used PCa as a model system for two case studies. Metabolic studies have identified significant changes in metabolites in both histidine and glycerophospholipid metabolism pathways, but gene expression changes in the original network models were unable to explain the observed metabolic differences. Our aim was to investigate if FunHoP could identify the possible changes in expression levels leading to the observed changes in metabolites. The dataset from TCGA was further used in the following case studies due to its high number of samples and thereby statistical power.

#### Case study 1: histidine metabolism

**Figure 3:**
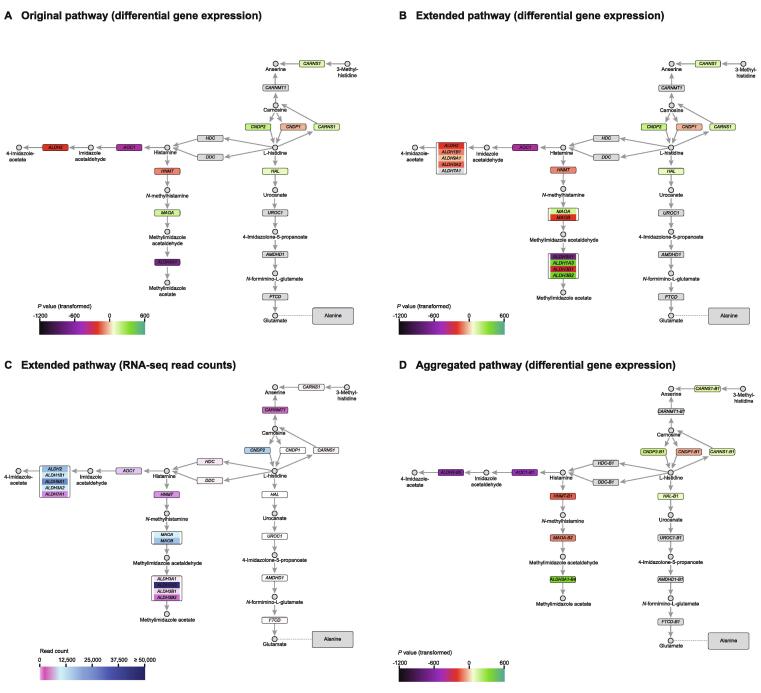
**Pathway of histidine metabolism** **A.** Original pathway colored by differential gene expression on a log-scale. **B.** Expanded pathway colored by differential gene expression. **C.** Expanded pathway colored by RNA-seq read counts. **D.** Aggregated pathway colored by differential gene expression at the node level.

The first case study looks at the histidine metabolism pathway. It has been shown that histidine is elevated in PCa compared to normal prostate tissue [29]. This elevation cannot be explained by differential changes in gene expression using the original pathway (Figure 3A). In the original pathway, histidine is produced from carnosine in two paths, by *CNDP2* or *CNDP1*. Histidine can then be converted back to carnosine through a loop by *CARNS1* (Figure 3A). Looking at the *P* values for the respective genes shows that *CNDP1* is downregulated, and *CNDP2* and *CARNS1* are up-regulated with *P* values within the same order of magnitude. Moreover, of the genes in the other paths leading away from histidine, *HAL* is up-regulated while *HDC* and *DDC* are unchanged (Figure 3A). Overall, this pathway is not compatible with the observed increase in histidine levels in PCa.

However, when using the FunHoP-expanded pathway to visualize all genes and nodes, we can see how the original pathway is an oversimplification of a more complex pathway including three nodes with multiple genes (Figure 3B). Many of these nodes have genes that are up- or down-regulated. As there are no functional homologs in the nodes most directly linked to histidine, expanding nodes alone does not lead to any improved interpretation in this case. However, when RNA-seq read counts are shown together with differential expression in the extended pathway, we are able to provide a possible explanation as to how histidine level may be elevated (Figure 3C).

For the paths leading to histidine synthesis, the most dominant gene in read counts is *CNDP2,* which is up-regulated and has about 11,000 reads. Up-regulation of *CNDP2* pushes carnosine conversion to histidine. The down-regulated *CNDP1* has close to zero read counts and can be ignored. *CARNS1*, responsible for the loop back towards carnosine, has less than 100 reads, and is probably less influential than *CNDP2*. We can therefore assume that up-regulation of the highly expressed *CNDP2* most likely leads to increased production of histidine. For the paths leading away from histidine, all genes in the path leading towards glutamate (including the up-regulated *HAL*) have close to zero read counts, and can be ignored. With *HDC* and *DDC* remaining unchanged, there is no net change in histidine consumption. Increased histidine production through the highly expressed *CNDP2* combined with ignorable changes in histidine consumption, leads to a possible explanation for how histidine accumulates. Moreover, the genes further downstream of histamine (*i.e.*, *HNMT* and *AOC1*) are down-regulated with higher read counts (2519 and 3636 read counts, respectively), creating a bottleneck in the influx/efflux balance, which can lead to further increase in histidine levels. The overall read counts in the pathway seems to push towards accumulation of histidine, which is not used further downstream in any direction, allowing a build-up of histidine to happen. The histidine pathway also shows examples of nodes with high difference in read counts between genes in the node. One example is the *ALDH3A1* node, where *ALDH1A3* dominates with 46,595 read counts, while the three remaining genes have less than 1000 reads each. This further strengthens the idea that the differential expression of the dominant gene will determine the overall expression of the node.

The conclusions from the expanded network are also evident in the aggregated network at the node level (Figure 3D), where *CNDP2* is clearly highlighted, especially when looking at the pathway styled with read counts. The aggregated network shows how nodes that appear to be up-regulated in the original network are shown to be down-regulated, and vice versa. Overall, FunHoP provides a more complete pathway analysis, and is able to give a more precise explanation on how histidine can be elevated in PCa.

#### Case study 2: glycerophospholipid metabolism

**Figure 4:**
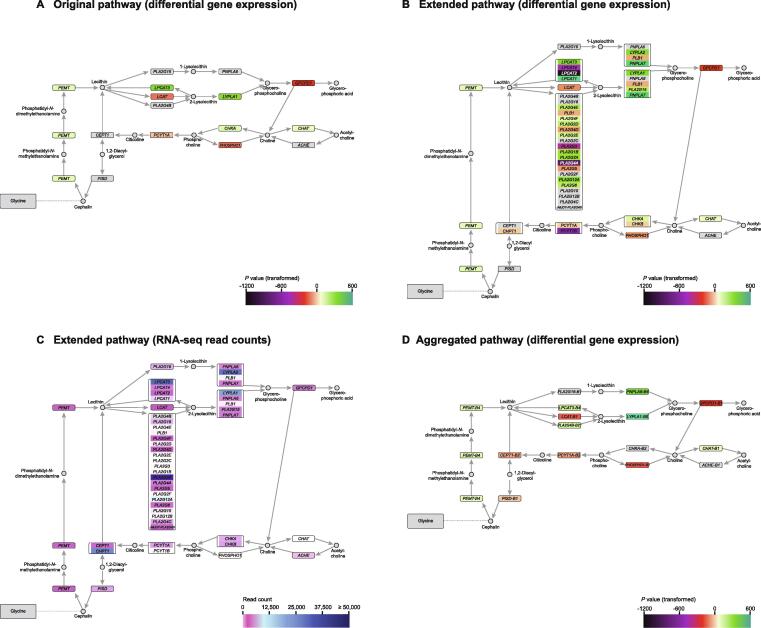
**Pathway of glycerophospholipid metabolism (part)** **A.** Original pathway colored by differential gene expression on a log-scale. **B.** Expanded pathway colored by differential gene expression. **C.** Expanded pathway colored by RNA-seq read counts **D.** Aggregated pathway colored by differential gene expression at the node level.

The second case study looks at part of the glycerophospholipid metabolism. The complete pathway is extensive and contains several complex nodes with up to 21 genes, which makes visualization and analysis challenging. Previous studies have shown elevated levels of GPC in PCa [30], [31], [32], and the original Cytoscape network from KEGG colored by differential expression is shown in Figure 4A.

The initial pathway does not provide an explanation to how GPC can be elevated based on differential gene expression values. In the reaction paths leading from lecithin towards GPC, three displayed genes are not significantly differentially expressed (*PLA2G16* and *PNPLA6* via 1-lysolecithin, and *PLA2G4B* towards 2-lysolecithin), and one is down-regulated (*LCAT* towards 2-lysolecithin), along with one up-regulated gene functioning in the opposite direction (up-regulated *LPCAT3* from 2-lysolecithin back to lecithin). This indicates that even if the conversion from 2-lysolecithin to GPC is up-regulated by *LYPLA1*, the reaction is just as much pushed away from 2-lysolecithin and back towards lecithin by *LPCAT3*, instead of towards GPC. Overall, this does not explain how GPC can be accumulated. However, when expanding the network, a more complex picture emerges, with more genes involved in several nodes and huge differences in RNA-seq read counts among genes and nodes (Figure 4B and C). The expanded networks provide a different interpretation of several nodes in the pathway.

A particularly complex node of 21 genes appears in the original *PLA2G4B* node. This node contains both non-significant genes and up-/down-regulated genes, with average RNA-seq read counts varying over many orders of magnitude. The clearly dominant gene is *PLA2G2A* with 71,482 read counts (Figure 4C), which is also up-regulated (Figure 4B). Since none of the other genes have read counts of comparable magnitude (the second highest is *PLA2G12A* with 4502 read counts), we see how this node changes from non-significant in the original network to up-regulated in the aggregated network (Figure 4D). The other nodes in the paths leading to production of GPC also show both up- and down-regulated genes. The most dominant gene in the *PNPLA6* node is *LYPLA2* (Figure 4C), which is up-regulated (Figure 4B). For the *LYPLA1* node, the dominant gene is *LYPLA1* (Figure 4C), which is up-regulated as in the original network (Figure 4A and B). Both dominant genes lead to their respective nodes being up-regulated in the aggregated network (Figure 4D), enabling two possible paths towards production of GPC. Both paths via 1-lysolecithin and 2-lysolecithin, respectively, are now up-regulated. Even though *PLA2G16* in the 1-lysolecithin path is not significant, *PLA2G16* still has 3246 reads, which indicates a flux through this path. For the path via 2-lysolecithin, the up-regulation of both the *PLA2G4B* and *LYPLA1* nodes reveals an unambiguous up-regulated path from lecithin to GPC. Though the *LPCAT3* node looping backwards upstream of GPC is also up-regulated, the pathway as a whole shows a net unambiguous flux towards GPC through several possible paths, explaining why GPC would accumulate in PCa. The expanded network also shows that *LCAT* (the sole gene of the LCAT node) has fewer reads than the *PLA2G4B* and *LPCAT3* nodes, making its down-regulation less important (Figure 4C).

Alongside providing more biological information, the GPC example also illustrates the possible complexity of nodes. With the full pathway having four highly complex multi-gene nodes of 21 genes, as well as several nodes with 4–6 genes, we see how difficult pathways can be to interpret. Using FunHoP, we show how we can gain important additional information by expanding the networks to show all genes, and by looking at differential expression and gene expression level simultaneously for network interpretation.

In order to validate our conclusions on the two case studies using data from TCGA, we performed the same analysis with the data from the Prensner cohort. Due to the generally smaller number of samples in the Prensner data, in addition to lower sequencing depth, many of the significant changes observed in TCGA were not statistically significant in Prensner. However, the overall patterns are also evident in both case studies, both in terms of the dominant genes within the pathway and the differential expression (Figures S1 and S2), which supports the conclusions on which genes can contribute to the elevated levels of histidine and GPC in PCa.

## Discussion

Metabolic pathway analysis is an important approach for analyzing gene expression. With the constantly growing amount of available data, we can improve our understanding of the complexity in biological systems, and continuously develop models to capture and utilize new data and information. However, the most commonly used pathway representations from databases and associated tools often give a simplified picture of metabolic pathways, focusing on only one gene in each network node, despite the fact that more genes may be able to perform the same enzymatic reaction. One example, which we have focused on in this study, is the current integration of KEGG and Cytoscape using KEGGScape.

We have therefore implemented a strategy for including all functional homologs of a gene in the analysis, based on the following assumptions:

First, we have to assume that the relevant genes in an expanded node indeed are functional homologs, *i.e*., with similar function. KEGG networks are manually curated, and documentation can be found within KEGG for genes, compounds, and reactions. When KEGGScape places a gene within a certain node, we assume that this gene is able to produce an enzyme that can catalyze the transition represented by the node. In FunHoP, we have implicitly made an assumption that the different genes within a node representing an enzymatic reaction also catalyze the reaction at a similar rate. This is a simplification, and to model the enzyme activity one should ideally also include enzyme efficiency and kinetics for the given situation. However, data on enzyme kinetics are usually not available, or very hard to obtain. We believe that our assumption on the enzyme activity correlating with expression level is at least reasonable for differences spanning several orders of magnitude, and represents a model improvement compared to networks where expression levels are not considered at all. Supporting this assumption is the observation that genes in a node usually belong to the same gene family. For example, for the node in the histidine metabolism pathway with *ALDH3A1* on top, all the other genes are aldehyde dehydrogenase paralogs that are able to catalyze the same reaction (Figure 2A).

Secondly, we have to assume that we actually can estimate relative expression levels of relevant genes. With microarrays being the previous gold standard to measure changes in gene expression, differential expression analysis and subsequent network mapping were limited to fold changes and *P* values. Variations in probe affinities made it difficult to assume anything about the real expression level differences between genes. However, with RNA-seq, one should be able to provide relative expression level measurements with much improved correlation to the real relative mRNA levels compared to microarrays.

Using the two assumptions on relative expression levels and similarity in enzyme efficiency described above, we can predict which of the genes is/are most likely to be responsible for a given reaction in a node. Especially for cases where the read-count difference for two genes in the same node spans several orders of magnitude, we find it likely that difference in expression level will take precedence over reaction efficiency. We have shown that read counts are highly reproducible for two independent patient cohorts for PCa. We observe that many pathway nodes typically consist of one or a few dominant genes in terms of expression level, supporting our claim that this is a highly relevant measure to include when evaluating the contribution from different enzymes in a node. For the single-gene nodes, the approach of looking at absolute gene expression can also reveal patterns in the pathways that are not evident from comparing *P* values alone. By using read counts, we are also capable of determining whether some paths are turned completely off, as in the case for the path leading from histidine to glutamine (Figure 3C).

A possible limitation of our approach is to which degree tissue-specific isoforms affect enzymatic activity and estimated expression levels of the genes represented in the nodes. Not all isoforms of a gene are necessarily enzymatically active. However, KEGG does not currently provide curated information on enzyme activity of isoforms. We have thus limited analysis to the gene level. However, an expansion to isoforms is conceptually possible within the FunHoP framework if such data become available. Another isoform related limitation is that genes with particularly short or long dominant isoforms compared to the canonical isoform model may lead to aberrant expression level estimation for the genes affected. In addition, tissue-specific isoform switches can potentially affect results from differential expression analysis. In this study, we have assumed that genes are presented by their canonical isoform.

The starting point for network analysis is usually an expression table with samples and genes, which for RNA-seq is presented as a table of read counts. It is thus preferable that the network analysis is reproducible with respect to RNA-seq RNA selection protocols, sequence length, library size, choice of alignment, and mapping tools. It was not possible to systematically investigate many settings (mostly due to lack of available data on prostate), but we demonstrate that the results are reproducible in two independent PCa cohorts with different properties. Both cohorts use poly-A selection of transcripts, but differ in sequence length, library size, and alignment/mapping tools.

We also assume that changes in transcript level are informative about changes in protein level. It is well known that a direct association between mRNA expression level, protein level, and subsequent protein activity is inaccurate, for example because of the effects of post-transcriptional and post-translational regulation of proteins on enzyme kinetics; however, the reasons in most cases are unknown. We cannot say with absolute certainty that an up-regulated pathway with multiple read counts will result in a similar increased number of metabolites. A study by Schwanhäusser et al. [33] shows a correlation between mRNA and protein copy numbers in NIH3T3 mouse fibroblasts, which was found to be 0.41. When considering translation rate constants, the correlation went up to 0.95.

Other studies in different organisms have also shown correlations, although this is organism dependent [32], [34]. FunHoP does not pretend to describe the complete picture, but still represents a significant improvement compared to analyses where all genes are assumed to have the same expression level, or where multiple genes in the same node are not taken into account at all.

In the KEGG database, histidine is also involved in two other pathways that can affect the overall levels of this metabolite. In aminoacyl-tRNA biosynthesis (KEGG: hsa00970), histidine is converted to L-histidyl-tRNA(his), catalyzed by *HARS* and *HARS2.* Neither of these genes show significant changes, which indicates that this does not affect the level of histidine between the samples. In beta-alanine metabolism (KEGG: hsa00910), histidine is involved in the same step as the one in our case study, although we here see a more complete picture of carnosine being converted into histidine and beta-alanine. This is performed by the same enzymes as in the case study (*CNDP1/CNDP2*). As we know, *CNDP1* is down-regulated and has close to zero read counts, and *CNDP2* is up-regulated with 11,217 read counts. This should indicate that beta-alanine is also elevated in PCa, which was confirmed by the same study [29]. Overall, we see how the case study provides a possible explanation on how histidine can be elevated in PCa, and our solution also fits with other available measurements of related metabolites [29].

GPC is also involved in another pathway: ether lipid metabolism (KEGG: hsa00565), where GPC can also be produced by conversion of 1-(1-alkenyl)-sn-glycero-3-phosphocholine by *TMEM86B*. However, this gene does not show a significant change between PCa tissue and normal tissue, and hence we can explain the elevated levels of GPC by the extracted part of glycerophospholipid metabolism shown in the case study. Another possibility for GPC to be elevated using the original network would be if the level of 2-lysolecithin was high, the up-regulated *LYPLA1* converted 2-lysolecithin to GPC. To our knowledge, 2-lysolecithin has not been documented as high in PCa.

Choline metabolism in PCa is a well-studied topic, especially in regard to relevant metabolites and identification of potential biomarkers [30], [31], [32], [35], [36]. The pathways involved in the metabolism are still not fully covered, and our findings from case study 2 are therefore of special interest. These results will be focused on in later studies.

The current version of FunHoP supports the human metabolic pathways found in KEGG, with exception of the glycan-related pathways (mostly found in “Glycan biosynthesis and metabolism”, category 1.7), which uses a different type of visualization (“lines” instead of the traditional “rectangles”). These lines cannot be colored and expanded similarly to gene nodes, and are hence not suitable for pathway analysis in Cytoscape. Another challenge with the glycan-related pathways is that many of the children lack reactions in the downloaded XML files, even if the genes are presented as rectangles, and hence parts of the networks seem to consist of random genes with no connection to the path. It is possible to extend and style these gene nodes like in other pathways, but the missing reactions will still be lost. These problems are due to the way KEGG builds the XML file and how the file is read by KEGGScape.

As seen in the Tables S1 and S2, a total of 64 out of the 71 pathways contain at least one multi-gene node. The 71 pathways contain a total of 1974 nodes, of which 768 has multiple genes. Even though this only accounts for 39% of all nodes, it still means that for 90% of the pathways there is a possibility that not all relevant data will be included in the analysis. As we have shown, a single multi-gene node can change the entire interpretation of the pathway when all genes are included. Having at least one multi-gene node for 90% of the metabolism-related pathways used in this study demonstrates the importance of developing tools like FunHoP.

The first part of FunHoP, which deals with expanding the nodes with multiple genes, could possibly be solved also with other KGML-readers. CyKEGGParser has similar functions where all the “hidden” genes get a new node, with its own edges. This displays all the genes within a node as separate nodes, and these nodes can be colored and analyzed similarly as the original ones. However, as this study has shown, there are some nodes that contain a very large number of genes, which makes the analysis and interpretation challenging without further filtering by read counts from RNA-seq. CyKEGGParser is a KGML-reader/tweaker and does not have any of the features of FunHoP with regard to using reads from RNA-seq to determine an overall expression value for all genes in a multi-gene node. KEGGScape is a pure KGML-reader, which allows for running the pathway XML files through FunHoP locally and using KEGGScape to import the improved files. KEGGScape does not bend the edges the way CyKEGGParser does, and does not separate the functional homologs when reading the KEGG XML files. However, CyKEGGParser has many useful features such as corrections of inconsistencies in pathways and tissue-specific tuning, and these features could be interesting to consider in future studies.

For the cases where a KEGG protein complex contains nodes with multiple genes, it is dealt with by adding an expanded node on top of the protein complex. All genes can hence be seen and colored by expression, although the user may have to do a bit of manual editing of the network. This is a challenge in visualization of the networks, as an expanded node will be placed in the same position as the original node, but in most cases, it takes up more space than what was originally allocated.

To improve FunHoP and make it easier for others to use it, solutions for the problems above are under development. Converting FunHoP into a Cytoscape app is also in development, which will make it easier for all users to apply this method to their own analyses.

## Conclusion

In this study, we have shown how FunHoP can be used to expand nodes from KEGG in Cytoscape to include all alternative genes present in a node. We have shown how *P* values from differential expression are not sufficient to determine regulation in a pathway, and how using the read counts from RNA-seq can facilitate metabolic network interpretation. Finally, we have shown that information in the extended networks can be aggregated to create more simplified networks at the node level, taking data from all genes into account.

By comparison of measured values of histidine and GPC in PCa and healthy prostate tissue from literature, we have shown how our analysis can explain why these metabolites are elevated, whereas the original pathway representations could not. We have also managed to show how differential expression based on *P* values does not differentiate between highly expressed genes and lowly expressed genes. By incorporating RNA-seq read counts into the analysis, we have highlighted genes that are highly expressed and more likely to dominate within a pathway. Overall, we show that FunHoP, by incorporating more biological information on network nodes and genes from KEGG, is able to provide improved pathway analysis.

## Code availability

Code and documentation for running FunHoP can be found at https://github.com/kjerstirise/FunHoP.

## CRediT author statement

**Kjersti Rise:** Conceptualization, Software, Validation, Formal analysis, Data curation, Writing - original draft, Writing - review & editing, Visualization. **May-Britt Tessem:** Writing - review & editing, Funding acquisition. **Finn Drabløs:** Conceptualization, Writing - review & editing, Supervision, Funding acquisition. **Morten B. Rye:** Conceptualization, Software, Formal analysis, Writing - review & editing, Visualization, Supervision, Funding acquisition. All authors have read and approved the final manuscript.

## Competing interests

The authors have declared no competing interests.

## Supplementary material

Supplementary File S1 **How FunHoP changes the XML files - node example**.

Supplementary Figure S1 **Pathway of histidine metabolism – Prensner. A**. Original pathway colored by differential gene expression from the Prensner cohort on a log-scale, ranging from –1200 (black) to 600 (dark green) via zero (yellow). The first impression shows close to none significant nodes. **B**. Expanding the nodes reveals multiple genes that show significant differential expression, which was not possible to see using the original network. **C**. Coloring the expanded network based on read counts, we see the same trends as in the network colored by the TCGA cohort. *CNDP2* is highly expressed, in a much higher degree than *CARNS1*, which changes histidine back to carnosine, and *HDC* and *DDC*, which converts histidine towards histamine. The path from histidine to glutamate contains close to zero read counts, which means histidine can be converted from carnosine, but has little conversion from histidine and onwards. We can also see how the counts are distributed within the multiple gene nodes, and predict how this will change the outcome of doing differential expression based on nodes as a total instead of single genes. **D**. The predictions from B can for instance be seen in the *ALDH3A1* node, where the upregulated *ALDH1A3* dominates the other genes when it comes to read counts, and hence the node becomes upregulated instead of downregulated. Despite the fact that *CNDP2* is still not significant, we can still see that there are multiple reads leading towards histidine, and very few leading away from it.

Supplementary Figure S2 **Pathway of glycerophosphocholine metabolism (part) – Prensner. A**. Original pathway colored by differential gene expression from the Prensner cohort on a log-scale, ranging from –1200 (black) to 600 (dark green) via zero (yellow). As for the histidine pathway, the number of significantly differentially expressed genes is lower than in TCGA. We can however see the same tendencies, with no particular path leading towards the formation and accumulation of glycerophosphocholine. **B**. Expanding the nodes reveals a few more genes that are differentially expressed. **C**. Coloring based on read counts reveals similar tendencies as with the TCGA cohort, for instance with *PLA2G2A* being the dominant gene within the *PLA2G4B* node. We can also see that the genes towards glycerophosphocholine contains many read counts. **D**. Despite there not being many significant genes after collapsing the nodes, the knowledge from the read counts still makes is plausible to assume how glycerophosphocholine is accumulated.
