# Supplementary material for: FunHoP: Enhanced Visualization and Analysis of Functionally Homologous Proteins in Complex Metabolic Networks
Source: Genomics Proteomics Bioinformatics. 2021 Mar 17;19(5):848–59. doi: 10.1016/j.gpb.2021.03.003 (PMC9170767; doi:10.1016/j.gpb.2021.03.003)
Supplement: Supplementary File S1 — How FunHoP changes the XML files - node example. [file mmc1.docx]

**File S1 How FunHoP changes the XML files - node example**

The child represents the *ALDH3A1* node from histidine metabolism (hsa00340). This multi-gene node contains the following genes: *ALDH3A1*, *ALDH1A3*, *ALDH3B1*, and *ALDH3B2*. Notice how the entry namestrings (green) and the graphics namestrings (red) change.

1. The original node

Notice how the entry namestring and the link (blue) contains IDs to all four genes within the node, but the graphics namestring contains *ALDH3A1* and then the names for *ALDH3A1* in other organisms.

<entry id="56" name="hsa:218 hsa:220 hsa:221 hsa:222" type="gene" reaction="rn:R04996"

link="<http://www.kegg.jp/dbget-bin/www_bget?hsa:218+hsa:220+hsa:221+hsa:222>">

<graphics name="ALDH3A1, ALDH3, ALDHIII..." fgcolor="#000000"

bgcolor="#BFFFBF" type="rectangle" x="574" y="520" width="46" height="17"/>

</entry>

2. Change the namestring

By using the IDs in the entry namestring in the original files, the graphics namestring can be changed to contain only the human name for all of the genes within the node.

<entry id="56" link="<http://www.kegg.jp/dbget-bin/www_bget?hsa:218+hsa:220+hsa:221+hsa:222>"

name="hsa:218 hsa:220 hsa:221 hsa:222" reaction="rn:R04996" type="gene">

<graphics bgcolor="#BFFFBF" fgcolor="#000000" height="17" name="ALDH3A1

ALDH1A3 ALDH3B1 ALDH3B2" type="rectangle" width="46" x="574" y="520" />

</entry>

3. Expanding the node

The node has been expanded by extracting all genes except the first one, and creating new children for them. The original child now contains only one ID and one name.

<entry id="56" link="<http://www.kegg.jp/dbget-bin/www_bget?hsa:218+hsa:220+hsa:221+hsa:222>"

name="hsa:218" reaction="rn:R04996" type="gene">

<graphics bgcolor="#BFFFBF" fgcolor="#000000" height="17" name="ALDH3A1"

type="rectangle" width="46" x="574" y="639" />

</entry>

The three remaining genes from the node now have their own, new entries. All the IDs and names are copied from the original node.

<entry id="132" link="<http://www.kegg.jp/dbget-bin/www_bget?hsa:218+hsa:220+hsa:221+hsa:222>"

name="hsa:220" reaction="rn:R04996" type="gene"><graphics bgcolor="#BFFFBF"

fgcolor="#000000" height="17" name="ALDH1A3" type="rectangle" width="46" x="574"

y="656" />

</entry>

<entry id="133"link="<http://www.kegg.jp/dbget-bin/www_bget?hsa:218+hsa:220+hsa:221+hsa:222>"

name="hsa:221" reaction="rn:R04996" type="gene"><graphics bgcolor="#BFFFBF"

fgcolor="#000000" height="17" name="ALDH3B1" type="rectangle" width="46" x="574"

y="673" />

</entry>

<entry id="134"link="<http://www.kegg.jp/dbget-bin/www_bget?hsa:218+hsa:220+hsa:221+hsa:222>"

name="hsa:222" reaction="rn:R04996" type="gene"><graphics bgcolor="#BFFFBF"

fgcolor="#000000" height="17" name="ALDH3B2" type="rectangle" width="46" x="574"

y="690" />

</entry>

When all the genes have new entries, the new entries can be combined with the original one in a group (light blue). Notice that the components of the group (purple) are connected by the entry IDs.

<entry id="135" name="undefined" type="group">

<graphics bgcolor="#BFFFBF" fgcolor="#000000" height="68" type="rectangle" width="55"

x="574" y="657.75" /> <component id="56" /> <component id="132" />

<component id="133"/><component id="134" />

</entry>

4. Collapsing the node

In order to visualize the collapsed read counts from all genes within a multi-gene node, the XML files need new names to match with the differential expression of the groups. Using the original XML files (from 1.), the number of IDs in the node is counted (here four), and the first name in the namestring is extracted and added "-Bx", where x represents the number of genes. This new name replaces the old namestring, and nodes can be colored using name as key.

<entry id="56" link="<http://www.kegg.jp/dbget-bin/www_bget?hsa:218+hsa:220+hsa:221+hsa:222>"

name="hsa:218 hsa:220 hsa:221 hsa:222" reaction="rn:R04996" type="gene">

<graphics bgcolor="#BFFFBF" fgcolor="#000000" height="17" name="ALDH3A1-B4"

type="rectangle" width="46" x="574" y="639" />

</entry>
