# Supplementary material for: FunHoP: Enhanced Visualization and Analysis of Functionally Homologous Proteins in Complex Metabolic Networks
Source: Genomics Proteomics Bioinformatics. 2021 Mar 17;19(5):848–59. doi: 10.1016/j.gpb.2021.03.003 (PMC9170767; doi:10.1016/j.gpb.2021.03.003)
Supplement: Supplementary Figure S1 — Pathway of histidine metabolism – Prensner. A. Original pathway colored by differential gene expression from the Prensner cohort on a log-scale, ranging from –1200 (black) to 600 (dark green) via zero (yellow). The first impression shows close to none significant nodes. B. Expanding the nodes reveals multiple genes that show significant differential expression, which was not possible to see using the original network. C. Coloring the expanded network based on read counts, we see the same trends as in the network colored by the TCGA cohort. CNDP2 is highly expressed, in a much higher degree than CARNS1, which changes histidine back to carnosine, and HDC and DDC, which converts histidine towards histamine. The path from histidine to glutamate contains close to zero read counts, which means histidine can be converted from carnosine, but has little conversion from histidine and onwards. We can also see how the counts are distributed within the multiple gene nodes, and predict how this will change the outcome of doing differential expression based on nodes as a total instead of single genes. D. The predictions from B can for instance be seen in the ALDH3A1 node, where the upregulated ALDH1A3 dominates the other genes when it comes to read counts, and hence the node becomes upregulated instead of downregulated. Despite the fact that CNDP2 is still not significant, we can still see that there are multiple reads leading towards histidine, and very few leading away from it. [file mmc2.pdf]

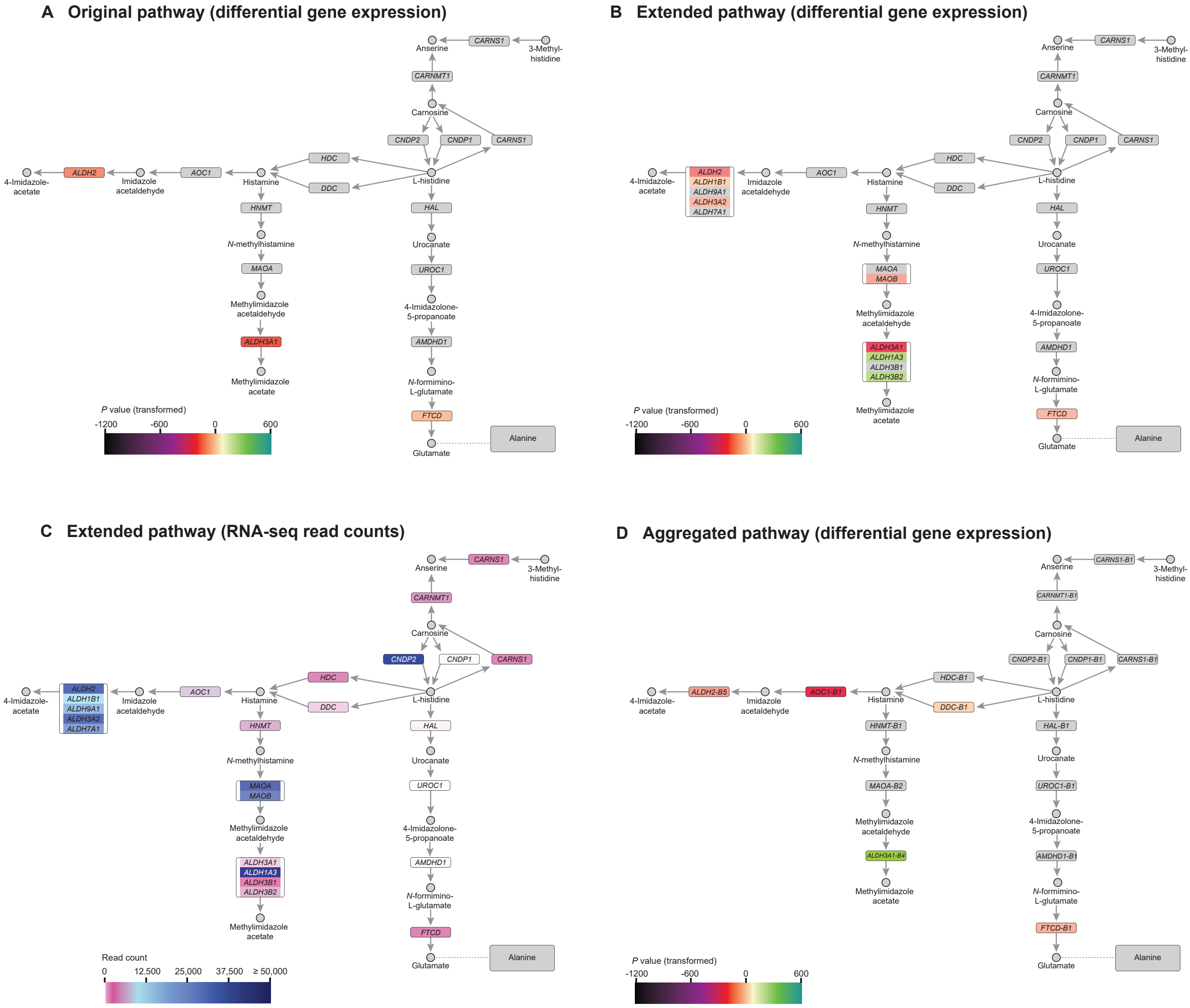
A Original pathway (differential gene expression)
B Extended pathway (differential gene expression)
C Extended pathway (RNA-seq read counts)
D Aggregated pathway (differential gene expression)
Anserine
CARNS1
3-Methyl-histidine
CARNMT1
Carnosine
CNDP2
CNDP1
L-histidine
HDC
DDC
Histamine
AOC1
Imidazole acetaldehyde
ALDH2
4-Imidazole-acetate
ALDH1B1
ALDH9A1
ALDH3A2
ALDH7A1
HNMT
N-methylhistamine
MAOA
MAOB
Methylimidazole acetaldehyde
ALDH3A1
ALDH1A3
ALDH3B1
ALDH3B2
Methylimidazole acetate
HAL
Urocanate
UROC1
4-Imidazolone-5-propanoate
AMDHD1
N-formimino-L-glutamate
FTCD
Glutamate
Alanine
P value (transformed)
-1200
-600
0
600
Read count
0
12,500
25,000
37,500
≥ 50,000
CARNS1-B1
CARNMT1-B1
CNDP2-B1
CNDP1-B1
HDC-B1
DDC-B1
AOC1-B1
ALDH2-B5
HNMT-B1
MAOA-B2
ALDH3A1-B4
HAL-B1
UROC1-B1
AMDHD1-B1
FTCD-B1
