# Supplementary material for: FunHoP: Enhanced Visualization and Analysis of Functionally Homologous Proteins in Complex Metabolic Networks
Source: Genomics Proteomics Bioinformatics. 2021 Mar 17;19(5):848–59. doi: 10.1016/j.gpb.2021.03.003 (PMC9170767; doi:10.1016/j.gpb.2021.03.003)
Supplement: Supplementary Figure S2 — Pathway of glycerophosphocholine metabolism (part) – Prensner. A. Original pathway colored by differential gene expression from the Prensner cohort on a log-scale, ranging from –1200 (black) to 600 (dark green) via zero (yellow). As for the histidine pathway, the number of significantly differentially expressed genes is lower than in TCGA. We can however see the same tendencies, with no particular path leading towards the formation and accumulation of glycerophosphocholine. B. Expanding the nodes reveals a few more genes that are differentially expressed. C. Coloring based on read counts reveals similar tendencies as with the TCGA cohort, for instance with PLA2G2A being the dominant gene within the PLA2G4B node. We can also see that the genes towards glycerophosphocholine contains many read counts. D. Despite there not being many significant genes after collapsing the nodes, the knowledge from the read counts still makes is plausible to assume how glycerophosphocholine is accumulated. [file mmc3.pdf]

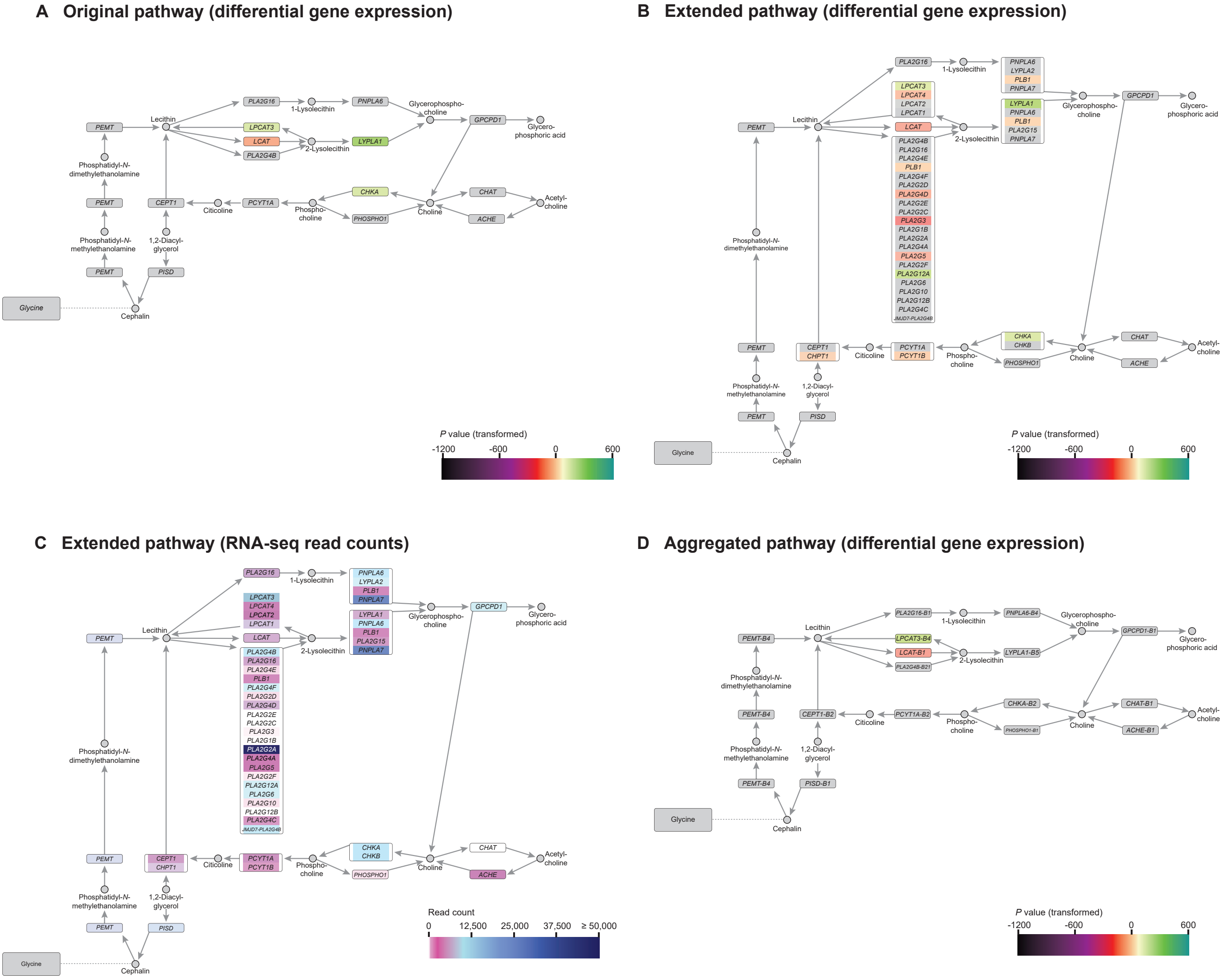
A Original pathway (differential gene expression)
B Extended pathway (differential gene expression)
C Extended pathway (RNA-seq read counts)
D Aggregated pathway (differential gene expression)
Lecithin
1-Lysolecithin
2-Lysolecithin
Glycerophospho-choline
Glycero-phosphoric acid
Phosphatidyl-N-dimethylethanolamine
Phosphatidyl-N-methylethanolamine
1,2-Diacyl-glycerol
Citicoline
Phospho-choline
Choline
Acetyl-choline
Cephalin
Glycine
PEMT
CEPT1
CHPT1
PISD
PCYT1A
PCYT1B
CHKA
CHKB
PHOSPHO1
CHAT
ACHE
GPCPD1
PLA2G16
PNPLA6
LYPLA1
LYPLA2
PLB1
PNPLA7
PLA2G15
LPCAT3
LPCAT4
LPCAT2
LPCAT1
LCAT
PLA2G4B
PLA2G4E
PLA2G4F
PLA2G2D
PLA2G4D
PLA2G2E
PLA2G2C
PLA2G3
PLA2G1B
PLA2G2A
PLA2G4A
PLA2G5
PLA2G2F
PLA2G12A
PLA2G6
PLA2G10
PLA2G12B
PLA2G4C
JMJD7-PLA2G4B
PEMT-B4
CEPT1-B2
PISD-B1
PCYT1A-B2
CHKA-B2
PHOSPHO1-B1
CHAT-B1
ACHE-B1
GPCPD1-B1
PLA2G16-B1
PNPLA6-B4
LPCAT3-B4
LCAT-B1
PLA2G4B-B21
LYPLA1-B5
P value (transformed)
-1200
-600
0
600
Read count
0
12,500
25,000
37,500
≥ 50,000
