# Supplementary material for: FunHoP: Enhanced Visualization and Analysis of Functionally Homologous Proteins in Complex Metabolic Networks
Source: Genomics Proteomics Bioinformatics. 2021 Mar 17;19(5):848–59. doi: 10.1016/j.gpb.2021.03.003 (PMC9170767; doi:10.1016/j.gpb.2021.03.003)
Supplement: Supplementary Table S1 [file mmc4.docx]

**Table S1 Pathways that contain multi-gene nodes**

| **Pathway** | **Affected node** |
| --- | --- |
| Alanine, aspartate, and glutamate metabolism | 9/32 |
| Alpha-linolenic acid metabolism | 4/9 |
| Amino sugar and nucleotide sugar metabolism | 16/43 |
| Arachidonic acid metabolism | 18/44 |
| Arginine and proline metabolism | 13/41 |
| Arginine biosynthesis | 6/13 |
| Ascorbate and aldarate metabolism | 2/5 |
| Beta-alanine metabolism | 5/24 |
| Butanoate metabolism | 9/14 |
| Butirosin and neomycin biosynthesis | 1/2 |
| Caffeine metabolism | 1/9 |
| Citrate cycle (TCA cycle) | 14/22 |
| Cyanoamino acid metabolism | 3/8 |
| Cysteine and methionine metabolism | 13/33 |
| D-Glutamine and D-glutamate metabolism | 3/3 |
| Drug metabolism - cytochrome P450 | 15/61 |
| Drug metabolism - other enzymes | 10/29 |
| Ether lipid metabolism | 11/19 |
| Fatty acid biosynthesis | 2/46 |
| Fatty acid degradation | 35/62 |
| Fatty acid elongation | 19/39 |
| Folate biosynthesis | 4/19 |
| Fructose and mannose metabolism | 10/19 |
| Galactose metabolism | 7/24 |
| Glutathione metabolism | 9/26 |
| Glycerolipid metabolism | 11/18 |
| Glycerophospholipid metabolism | 25/49 |
| Glycine, serine, and threonine metabolism | 11/36 |
| Glycolysis / Gluconeogenesis | 20/35 |
| Histidine metabolism | 3/17 |
| Inositol phosphate metabolism | 18/37 |
| Linoleic acid metabolism | 3/12 |
| Lysine degradation | 10/25 |
| Metabolism of xenobiotics by cytochrome P450 | 34/86 |
| Nicotinate and nicotinamide metabolism | 13/25 |
| Nitrogen metabolism | 2/4 |
| One carbon pool by folate | 9/22 |
| Oxidative phosphorylation | 26/112 |
| Pantothenate and CoA biosynthesis | 6/15 |
| Pentose and glucuronate interconversions | 6/14 |
| Pentose phosphate pathway | 8/25 |
| Phenylalanine metabolism | 4/11 |
| Phenylalanine, tyrosine, and tryptophan biosynthesis | 2/7 |
| Porphyrin and chlorophyll metabolism | 6/21 |
| Propanoate metabolism | 11/24 |
| Purine metabolism | 51/107 |
| Pyrimidine metabolism | 37/66 |
| Pyruvate metabolism | 14/23 |
| Retinol metabolism | 25/36 |
| Selenocompound metabolism | 7/14 |
| Sphingolipid metabolism | 19/33 |
| Starch and sucrose metabolism | 13/25 |
| Steroid biosynthesis | 3/35 |
| Steroid hormone biosynthesis | 44/107 |
| Sulfur metabolism | 6/11 |
| Synthesis and degradation of ketone bodies | 5/5 |
| Taurine and hypotaurine metabolism | 4/8 |
| Terpenoid backbone biosynthesis | 8/18 |
| Tryptophan metabolism | 18/41 |
| Tyrosine metabolism | 16/46 |
| Ubiquinone and other terpenoid-quinone biosynthesis | 2/14 |
| Valine, leucine, and isoleucine biosynthesis | 4/4 |
| Valine, leucine, and isoleucine degradation | 22/49 |
| Vitamin B6 metabolism | 3/12 |
