# Supplementary material for: FunHoP: Enhanced Visualization and Analysis of Functionally Homologous Proteins in Complex Metabolic Networks
Source: Genomics Proteomics Bioinformatics. 2021 Mar 17;19(5):848–59. doi: 10.1016/j.gpb.2021.03.003 (PMC9170767; doi:10.1016/j.gpb.2021.03.003)
Supplement: Supplementary Table S2 [file mmc5.docx]

**Table S2 Pathways that do not contain any multi-gene nodes**

| **Pathway** | **Affected node** |
| --- | --- |
| Biotin metabolism | 0/11 |
| D-arginine and D-ornithine metabolism | 0/2 |
| Lipoic acid metabolism | 0/5 |
| Lysine biosynthesis | 0/4 |
| Primary bile acid biosynthesis | 0/47 |
| Riboflavin metabolism | 0/4 |
| Thiamine metabolism | 0/5 |
